# CircCNIH4 inhibits gastric cancer progression via regulating DKK2 and FRZB expression and Wnt/β-catenin pathway

**DOI:** 10.1186/s40709-021-00140-x

**Published:** 2021-08-07

**Authors:** Qi Shi, Chuanwen Zhou, Rui Xie, Miaomiao Li, Peng Shen, Yining Lu, Shijie Ma

**Affiliations:** Department of Gastroenterology, The Affiliated Huaian No. 1 People’s Hospital of Nanjing Medical University, No. 1, West Huanghe Road, Huaian, 223300 Jiangsu China

**Keywords:** CircCNIH4, Gastric cancer, DKK2, FRZB, Wnt/β-catenin pathway

## Abstract

**Background:**

Circular RNAs (circRNAs) have been reported to play an important role in tumor progression in various cancer types, including gastric cancer. The aim of this study was to investigate the role of circCNIH4 (hsa_circ_0000190) in gastric cancer and the underlying mechanism.

**Methods:**

The expression levels of circCNIH4 and Wnt antagonist genes were detected by quantitative real-time polymerase chain reaction (qRT-PCR). The protein levels of β-catenin, Ki67, Dickkopf 2 (DKK2) and Frizzled related protein (FRZB) were measured by western blot. Ectopic overexpression or knockdown of circCNIH4, proliferation, apoptosis, migration and invasion by 3-(4,5-dimethyl-2-thiazolyl)-2,5-diphenyl-2-H-tetrazolium bromide (MTT), flow cytometry and transwell assay in vitro, and in vivo experiment, were employed to assess the role of circCNIH4 in gastric cancer.

**Results:**

CircCNIH4 was downregulated in gastric cancer tissues and cells. Overexpression of circCNIH4 inhibited gastric cancer cell proliferation, migration and invasion and promoted apoptosis by inactivating Wnt/β-catenin pathway in vitro. CircCNIH4 induced the expression of DKK2 and FRZB in gastric cancer cells. Moreover, silencing of DKK2 or FRZB reversed circCNIH4 overexpression-mediated effects on gastric cancer cells. Additionally, circCNIH4 suppressed tumor growth via regulating DKK2 and FRZB expression in gastric cancer in vivo.

**Conclusion:**

Our study demonstrated that circCNIH4 played a tumor-inhibiting role through upregulating DKK2 and FRZB expression and suppressing Wnt/β-catenin pathway in gastric cancer, which might provide a potential biomarker for the diagnosis and treatment of gastric cancer.

**Supplementary Information:**

The online version contains supplementary material available at 10.1186/s40709-021-00140-x.

## Background

Gastric cancer is one of the most prevalent malignant tumors worldwide, and the morbidity and mortality of gastric cancer are still increasing [1, 2]. For the present, surgery is the only radical therapy for gastric cancer, and it can achieve a good effect on gastric cancer patients in early stage [3]. Although many efforts and improvements have been made in the diagnosis and therapy of gastric cancer, the prognosis of advanced gastric cancer patients is still poor. Therefore, it is very important to identify available targets for early diagnosis and therapy of gastric cancer.

Circular RNAs (circRNAs) are a kind of endogenous non-coding RNAs. Unlike linear RNAs, circRNAs have closed-loop structures without 3′ polyadenylated tails and 5′ caps, and this structure makes them more stable [4]. Increasing evidence has indicated that circRNAs play vital roles in the progression of various types of cancer, including gastric cancer [5, 6]. For example, circHECTD1 contributes to the development of gastric cancer via regulating the miR-1256/USP5 expression and β-catenin/c-Myc pathway [7]. Knockdown of hsa_circ_0008035 represses gastric cancer cells proliferation and invasion [8]. In addition, certain circRNAs can serve as biomarkers for the diagnosis of gastric cancer [9]. CircCNIH4, also named circ_0000190, was revealed to associate with tumor progression and can be a diagnostic target in gastric cancer [10]. However, the role of circCNIH4 in gastric cancer and the molecular mechanism remains largely unknown.

Dickkopf 2 (DKK2), an inhibiting factor of the Wnt pathway, is reported to be a crucial regulator in multiple cancers [11–13]. DKK2 is upregulated and predicates poor prognosis in pancreatic ductal adenocarcinoma [14], while in gastric cancer, DKK2 serves as a tumor-suppressor gene [15]. Frizzled related protein (FRZB), also known as secreted frizzled-related protein 3 (SFRP3), is manifested to involve in cancer development [16, 17]. It is also identified as a prognostic factor for patients with gastric cancer [18]. In addition, a previous study displayed that knockdown of FRZB promoted cell growth through Wnt/β-catenin pathway in gastric cancer [19]. However, the regulatory mechanism between circHECTD1 and DKK2 or FRZB has not been elucidated in gastric cancer.

In our study, we detected the expression of circHECTD1 in gastric cancer tissues and cells and explored its effects on Wnt/β-catenin pathway and tumor progression in gastric cancer cells. Moreover, we selected two Wnt antagonist genes, DKK2 and FRZB, which were upregulated by circHECTD1, and the regulatory mechanism was further studied. Additionally, the impact of circHECTD1 on gastric cancer in vivo was also investigated.

## Methods

### Clinical samples

Tissue samples were collected from 32 patients diagnosed with gastric cancer in The Affiliated Huaian No. 1 People’s Hospital of Nanjing Medical University, and gastric cancer tissues and adjacent normal tissues were collected through surgical resection. All patients signed informed consents and had no other treatment before surgery. This study obtained the approval of the Ethics Committee of The Affiliated Huaian No. 1 People’s Hospital of Nanjing Medical University.

### Cell culture

Human normal gastric mucosal epithelial cell line (GES-1) and human gastric cancer cell lines (MKN-74, HGC-27 and AGS) were bought from BeNa Culture Collection (Beijing, China). Cells were cultured in Roswell Park Memorial Institute (RPMI) 1640 medium (Gibco, Carlsbad, CA, USA) with 10% fetal bovine serum (FBS, Gibco, Carlsbad, CA, USA), and maintain the conditions of 37 °C and 5% CO_2_.

### RNA extraction and RNAse R treatment

Total RNA was isolated using TRIzol Reagent (Invitrogen, Carlsbad, CA, USA) following manufacturer instructions. Cytoplasmic and Nuclear RNA Purification Kit (Norgen Biotek Corp, Ontario, Canada) was used to extract cytoplasmic and nuclear RNA. For RNAse R treatment, total RNA (5 μg) was incubated with 20 U RNAse R (Epicentre Biotechnologies, Madison, WI, USA) for 1 h at 37 °C.

### Quantitative real-time polymerase chain reaction (qRT-PCR)

RNA was reversely transcribed into cDNA by PrimeScript RT Reagent Kit (Takara, Dalian, China). And then, qRT-PCR was performed with SYBR Green Master Mix (Takara) in accordance with the instructions. Glyceraldehyde-3-phosphate dehydrogenase (GAPDH) and U6 were used as internal controls, and the expression level was calculated by 2^−∆∆Ct^ method. The primers were listed in Table 1.

**Table 1:** The primer sequences used for qRT-PCR

Gene	Sequence (5′ to 3′)
circCNIH4	F: GAGGGCAGCTGAAGTCACAC
	R: ACCAGTGCAATGACATGAGC
CNIH4	F: TCAACTTACCTGTTGCCACTTG
	R: TCTGTTGGATCAAACACTCCCA
SFRP1	F: ACGTGGGCTACAAGAAGATGG
	R: CAGCGACACGGGTAGATGG
SFRP2	F: ACGGCATCGAATACCAGAACA
	R: CTCGTCTAGGTCATCGAGGCA
SFRP4	F: CCTGGAACATCACGCGGAT
	R: CGGCTTGATAGGGTCGTGC
DKK1	F: GTGCAAATCTGTCTCGCCTG
	R: GCACAACACAATCCTGAGGC
DKK2	F: GATGATCCTTGGTGGGGAC
	R: GTACCAAGGACTGGCATTCG
DKK4	F: TGGACTTCAACAACATCAGGAG
	R: GGTATTGCAGTCCGTGTCAG
IDAX	F: CTCATCAACTGTGGCGTCTG
	R: TTAGTTTGCCCTTCATTTCC
FRZB	F: ACGGGACACTGTCAACCTCT
	R: CGAGTCGATCCTTCCACTTC
U6	F: CTCGCTTCGGCAGCACA
	R: AACGCTTCACGAATTTGCGT
GAPDH	F: ACAACTTTGGTATCGTGGAAGG
	R: GCCATCACGCCACAGTTTC

### Vector construction and cell transfection

For the overexpression of circCNIH4, human circCNIH4 cDNA was cloned and inserted into the pcDNA3.1 (Invitrogen, Carlsbad, CA, USA), and the empty vector was severed as control (NC). Small interfering RNA (siRNA) against circCNIH4, DKK2 and FRZB (si-circCNIH4, si-DKK2 and si-FRZB) and the control (si-NC) were obtained from GenePharma (Shanghai, China). Lipofectamine 2000 (Invitrogen, Carlsbad, CA, USA) was used for transfection of vector or siRNA. For stable transfection, circCNIH4 cDNA was inserted into the PLCDH-ciR lentivirus expression vector (Geneseed, Guangzhou, China) and infected into MKN-74 cells. The cells were selected with 0.8 μg mL^−1^ puromycin.

### Western blot analysis

A protein extraction kit (Beyotime, Shanghai, China) was used to extract total protein. Then protein was separated by sodium dodecyl sulfate–polyacrylamide gel electrophoresis (SDS-PAGE) and transferred onto a polyvinylidene difluoride (PVDF) membrane (Millipore, Billerica, MA, USA). 5% nonfat milk was used to block the membrane, and then the membrane was incubated with the primary antibodies against β-catenin (1:5000; ab32572, Abcam, Cambridge, UK), Ki67 (1:5000; ab92742, Abcam, Cambridge, UK), DKK2 (1:1000; ab95274, Abcam, Cambridge, UK), FRZB (1:1000; ab205284, Abcam, Cambridge, UK) or GAPDH (1:5000; ab181602, Abcam, Cambridge, UK) overnight at 4 °C. Subsequently, the membrane was incubated with the secondary antibody (1:5000; ab205718, Abcam, Cambridge, UK) at room temperature for 1.5 h. The protein signals were showed by enhanced chemiluminescence reagents (Millipore, Billerica, MA, USA).

### Cell proliferation assay

Cell proliferation was measured by 3-(4,5-dimethyl-2-thiazolyl)-2,5-diphenyl-2-H-tetrazolium bromide (MTT) assay. Cells were cultured in a 96-well plate for 24 h after transfection. Then each well was added with 20 µL of MTT (5 mg mL^−1^) to incubate the cells for 4 h. Subsequently, cells were collected and added with 150 µL of dimethyl sulfoxide for the dissolution of formazan crystals. The absorbance at 490 nm wavelength was measured using a microplate reader (Thermo Fisher Scientific, Waltham, MA, USA).

### Flow cytometry assay

Apoptosis was determined according to annexin V-fluorescein isothiocyanate (V-FITC)/propidium iodide (PI) apoptosis kit (BD Bioscience, San Diego, CA, USA) instructions. Cells were collected after transfection for 48 h and then added annexin V-FITC and PI. After incubation for 20 min in a dark condition, cell apoptosis was tested by flow cytometry (BD Bioscience, San Diego, CA, USA).

### Transwell assay

Cell migration and invasion were detected with transwell chambers (Corning, Tewksbury, MA, USA) coated without and with Matrigel (BD Biosciences, San Diego, CA, USA), respectively. Cells with serum-free medium were added into the upper chamber, and the basolateral chamber was added cell medium with 10% serum. After cultured for 24 h, the migrated or invaded cells adhering to the lower surface of the membrane were fixed by 4% paraformaldehyde and dyed with 0.5% crystal violet solution. The stained cells were counted under a microscope (Thermo Fisher Scientific, Waltham, MA, USA).

### In vivo tumor formation assay

The animal experiment was approved by the Animal Care Committee of The Affiliated Huaian No. 1 People’s Hospital of Nanjing Medical University. 5-week-old nude mice were randomly divided into two groups (circCNIH4 overexpression group and negative control (NC, transfected with empty vectors) group. MKN-74 cells stably transfected with circCNIH4 overexpression vector or empty vector were subcutaneously injected into nude mice. After 10 days for injection, tumor length and width were gauged every 4 days. After 34 days, the tumor tissues were resected and weighed. Tumor volume was computed using the formula length × width^2^/2.

### Statistical analysis

Data were presented as mean ± standard deviation and analyzed by SPSS 22.0 (IBM Corp., Armonk, NY, USA). Each experiment was completed with at least three repeats. Differences between two groups or multiple groups were compared by Student’s *t*-test or One-Way Analysis of Variance (ANOVA). *p* < 0.05 was considered statistically significant.

## Results

### CircCNIH4 was downregulated in gastric cancer and mainly localized at cytoplasm

**Fig. 1:**
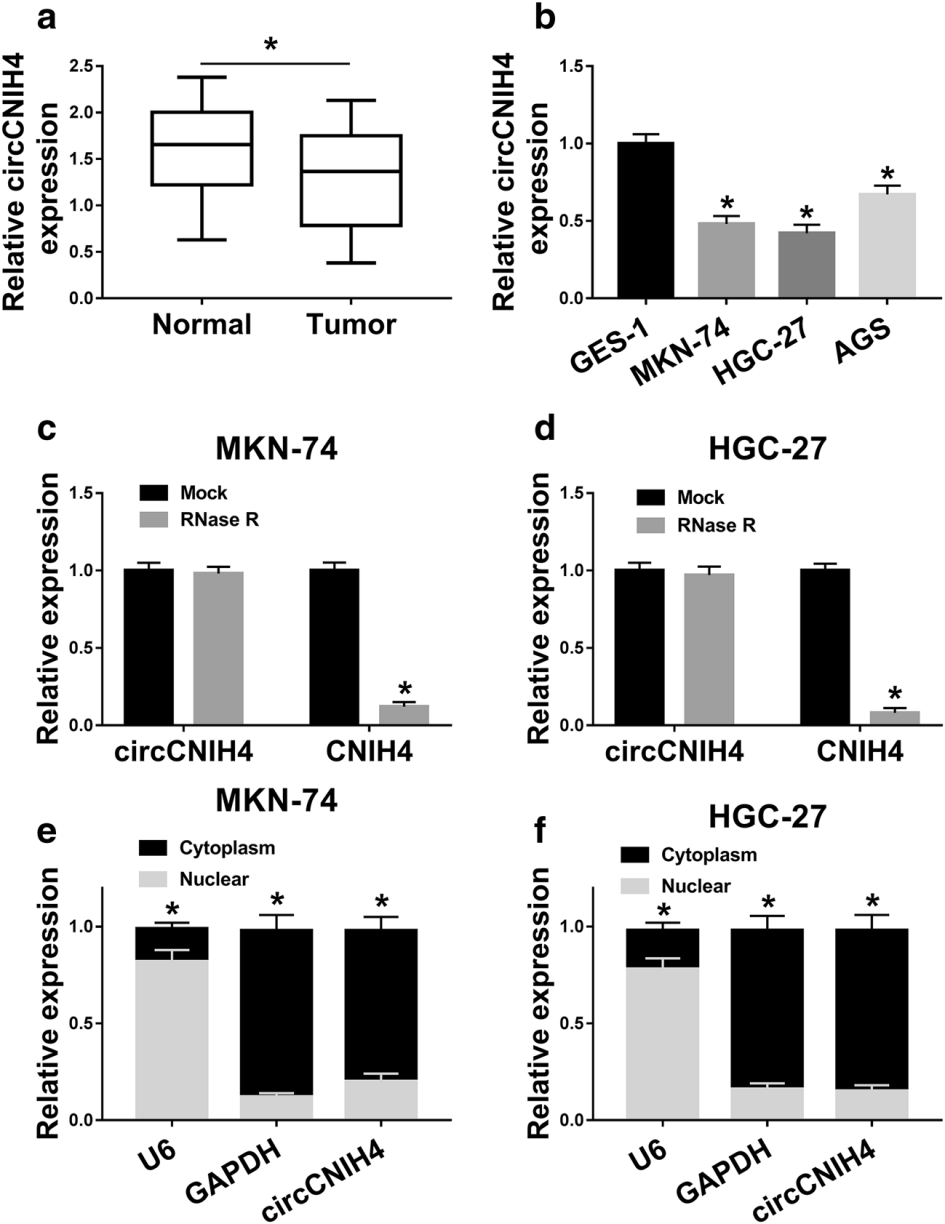
CircCNIH4 was downregulated in gastric cancer and mainly localized at cytoplasm. **a** The expression of circCNIH4 was detected by qRT-PCR in normal and gastric cancer tissues. **b** The expression of circCNIH4 was detected by qRT-PCR in normal cell line (GES-1) and three gastric cancer cell lines (MKN-74, HGC-27and AGS). **c** and **d** The expression of circCNIH4 and linear-CNIH4 (CNIH4) was measured by qRT-PCR with RNase R treated RNA in MKN-74 and HGC-27 cells. **e** and **f** The expression of circCNIH4 was detected by qRT-PCR in cytoplasm and nuclear of MKN-74 and HGC-27 cells. *p < 0.05

The expression of circCNIH4 was detected in gastric cancer, and it was found that circCNIH4 level was significantly reduced in gastric cancer tissues compared with normal tissues (Fig. 1a). Also, the expression of circCNIH4 was lower in three human gastric cancer cell lines (MKN-74, HGC-27 and AGS), especially in MKN-74 and HGC-27 cells than that in GES-1 cells (Fig. 1b). Thus, MKN-74 and HGC-27 cell lines were employed for subsequent experiments. To verify the circular structure of circCNIH4, the specific convergent and divergent primers that specifically amplified circCNIH4 or GAPDH were designed. Our results indicated that the convergent primers could amplify both circCNIH4 and GAPDH using cDNA and genomic DNA (gDNA) templates from gastric cancer cells (MKN-74 and HGC-27), while the divergent primers could only amplify circCNIH4 using cDNA as template rather than gDNA template (Additional file 1: Figure S1a). Next, Sanger sequencing was employed to directly verify the splice junction of circCNIH4 (Additional file 1: Figure S1b). Then, the RNA was treated with RNase R and performed qRT-PCR assay to evaluate its resistance to exonuclease. The result indicated that linear-CNIH4 (CNIH4) was digested by RNAse R while circCNIH4 could resist the digestion of RNAse R in both MKN-74 and HGC-27 cells (Fig. 1c, d). In addition, we explored the localization of circCNIH4 in MKN-74 and HGC-27 cells, and the qRT-PCR results showed that the circCNIH4 was highly expressed in cytoplasm rather than nuclear (Fig. 1e, f). These results implied that circCNIH4 might play a role in gastric cancer and mainly perform its function in cytoplasm.

### Overexpression of circCNIH4 suppressed proliferation, migration and invasion and enhanced apoptosis in gastric cancer cells via inactivating Wnt/β-catenin pathway

**Fig. 2:**
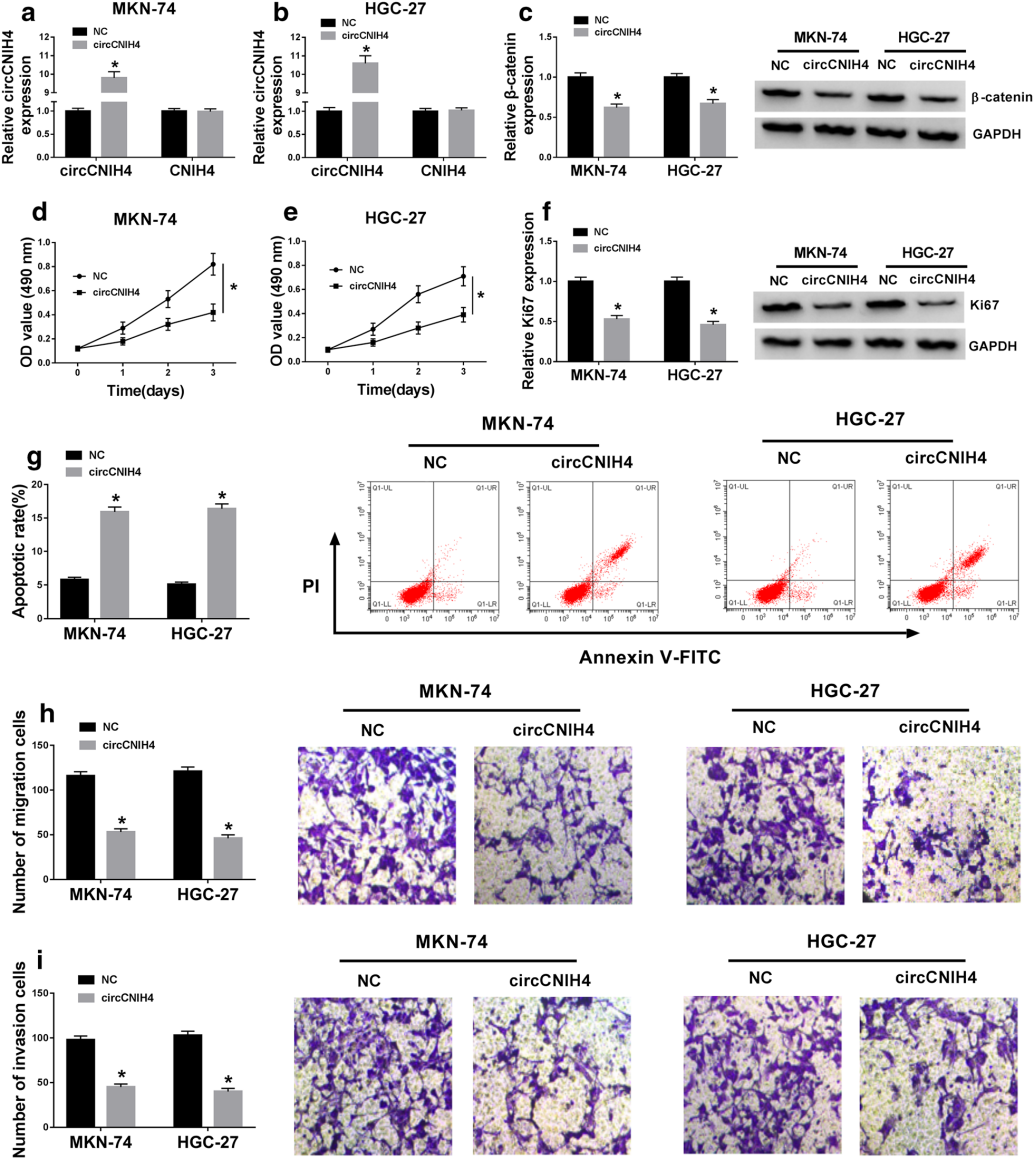
Overexpression of circCNIH4 suppressed gastric cancer cells progression via inactivating Wnt/β-catenin pathway. MKN-74 and HGC-27 cells were transfected with circCNIH4 overexpression vector or NC vector. **a** and **b** The expression of circCNIH4 was detected by qRT-PCR in MKN-74 and HGC-27 cells. **c** the protein level of β-catenin was measured by western blot in MKN-74 and HGC-27 cells. **d** and **e** Proliferation of MKN-74 and HGC-27 cells was determined by MTT assay. **f** The protein level of Ki67 was measured by western blot in MKN-74 and HGC-27 cells. **g** Apoptosis of MKN-74 and HGC-27 cells was detected by flow cytometry assay. **h** and **i** Migration and invasion of MKN-74 and HGC-27 cells were assessed by transwell assay. *p < 0.05

To investigate the role of circCNIH4 in gastric cancer, we overexpressed circCNIH4 in MKN-74 and HGC-27 cells. As shown in Fig. 2a, b, the expression of circCNIH4 was markedly increased in MKN-74 and HGC-27 cells transfected with circCNIH4 and the level of CNIH4 had no change. Then the expression of β-catenin was detected, and the result showed that the overexpression of circCNIH4 restrained the expression of β-catenin (Fig. 2c). This data revealed that circCNIH4 overexpression repressed Wnt/β-catenin pathway. MTT assay indicated that proliferation of MKN-74 and HGC-27 cells was impeded by overexpression of circCNIH4 (Fig. 2d, e). Ki67, a proliferation-related protein, was also inhibited in MKN-74 and HGC-27 cells transfected with circCNIH4 (Fig. 2f). In addition, flow cytometry assay displayed that apoptosis was promoted by overexpression of circCNIH4 in MKN-74 and HGC-27 cells (Fig. 2g). Subsequently, cell migration and invasion were determined, and the result indicated that overexpression of circCNIH4 inhibited migration and invasion of MKN-74 and HGC-27 cells (Fig. 2h, i). These results suggested that circCNIH4 overexpression suppressed the development of gastric cancer by inactivating Wnt/β-catenin pathway in vitro.

### CircCNIH4 promoted the expression of DKK2 and FRZB in gastric cancer cells

**Fig. 3:**
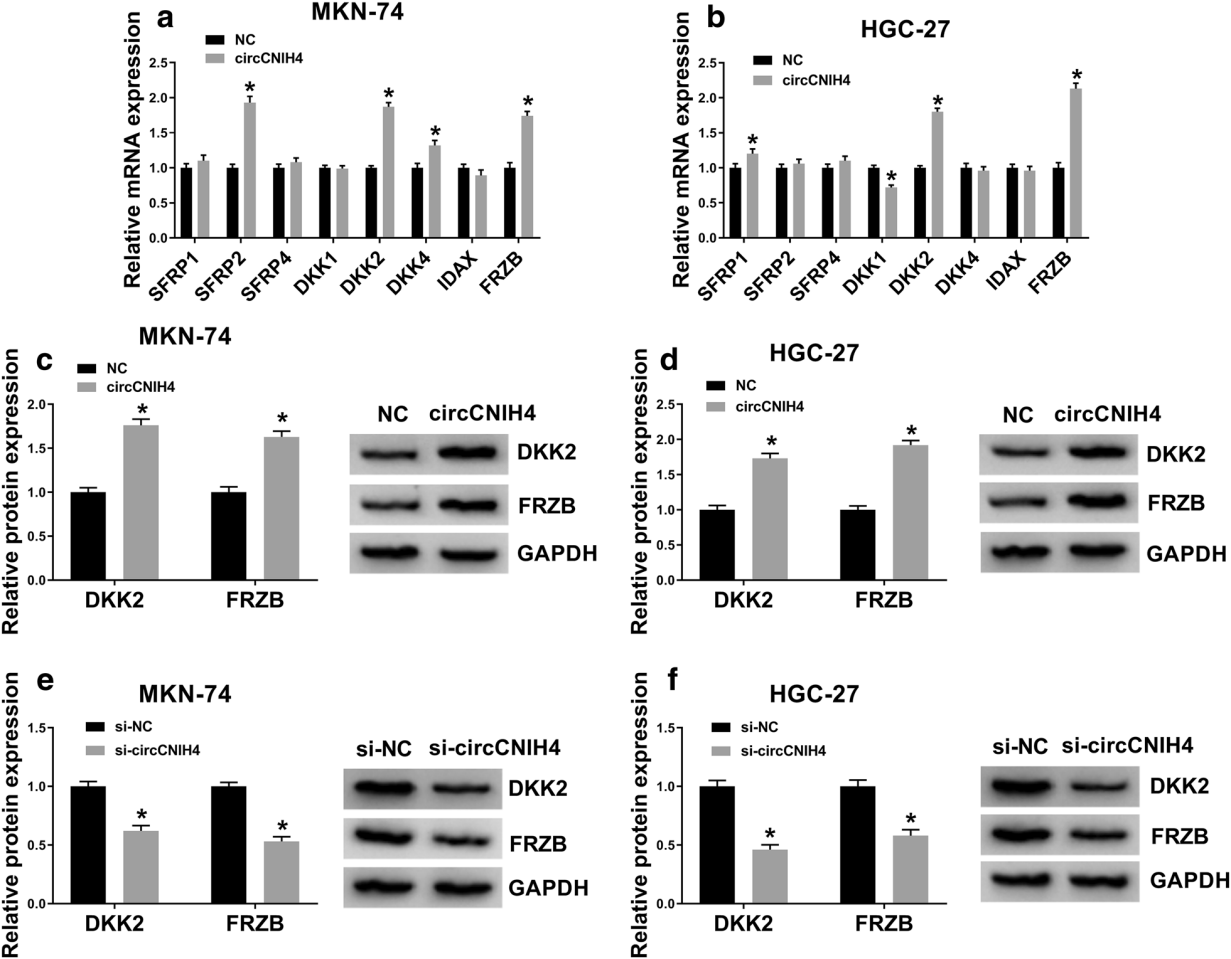
CircCNIH4 inhibited the expression of DKK2 and FRZB in MKN-74 and HGC-27 cells. **a** and **b** The mRNA levels of Wnt antagonist genes were detected by qRT-PCR in MKN-74 and HGC-27 cells transfected with circCNIH4 or NC (empty vectors). **c** and **d** The protein levels of DKK2 and FRZB were measured by western blot in MKN-74 and HGC-27 cells transfected with circCNIH4 or NC. **e** and **f** The protein levels of DKK2 and FRZB were measured by western blot in MKN-74 and HGC-27 cells transfected with si-circCNIH4 or si-NC. *p < 0.05

To explore the molecular mechanism of circCNIH4 on regulating Wnt/β-catenin pathway, the mRNA levels of Wnt/β-catenin pathway-related proteins were measured in MKN-74 and HGC-27 cells transfected with circCNIH4. The results revealed that DKK2 and FRZB were remarkably upregulated by circCNIH4 overexpression in both MKN-74 and HGC-27 cells (Fig. 3a, b). Moreover, the protein levels of DKK2 and FRZB were also significantly increased after circCNIH4 was overexpressed in MKN-74 and HGC-27 cells (Fig. 3c, d). Additionally, knockdown of circCNIH4 decreased the protein levels of DKK2 and FRZB in MKN-74 and HGC-27 cells (Fig. 3e, f). These findings suggested that circCNIH4 positively regulated the expression of DKK2 and FRZB in gastric cancer cells.

### Silencing of DKK2 reversed circCNIH4-mediated effects on gastric cancer cells

**Fig. 4:**
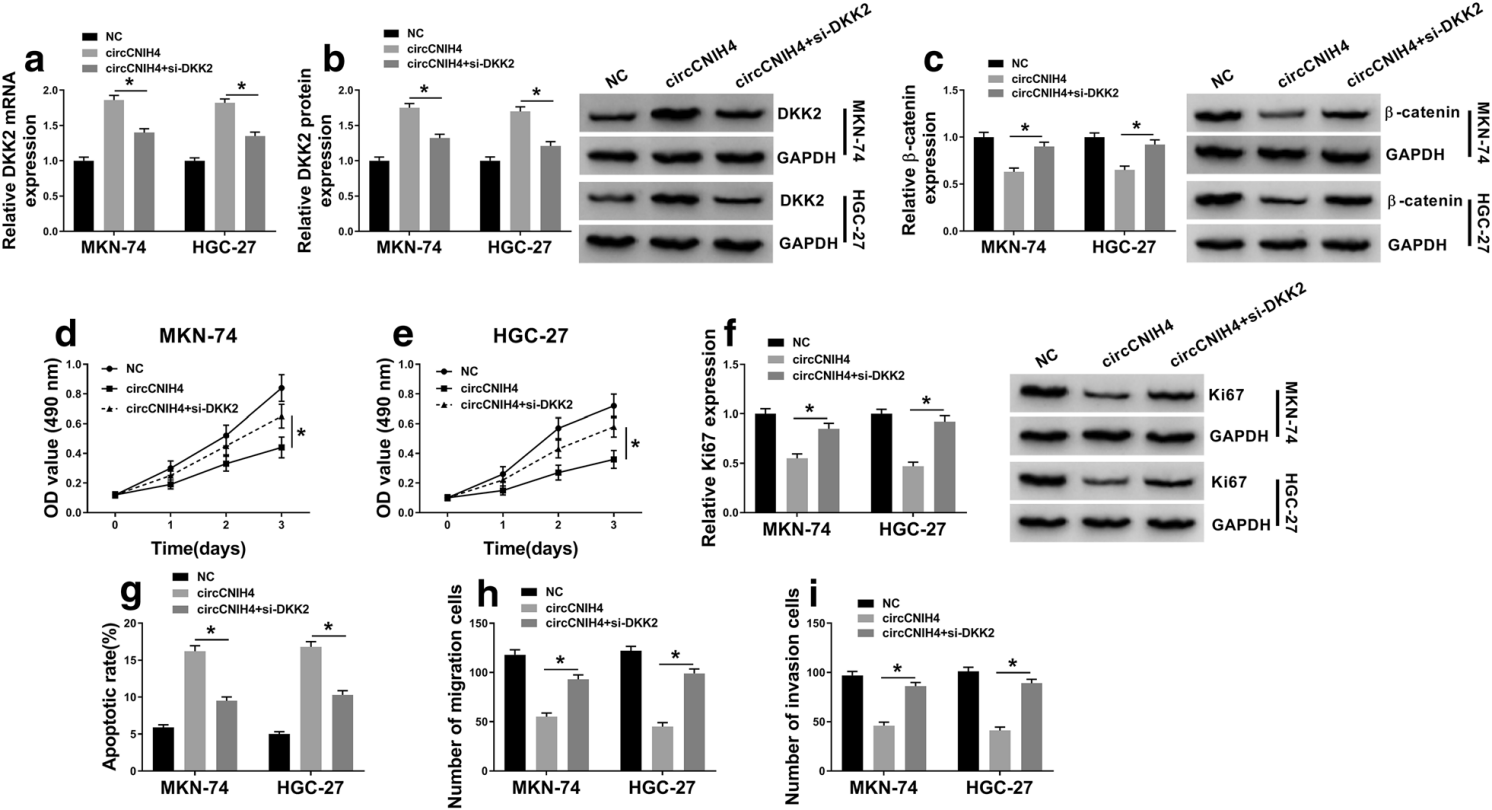
Silencing of DKK2 restored the effects of circCNIH4-mediated in gastric cancer cells. MKN-74 and HGC-27 cells were transfected with circCNIH4 or circCNIH4 + si-DKK2. **a** and **b** The mRNA and protein levels of DKK2 were detected in MKN-74 and HGC-27 cells. **c** The protein level of β-catenin was measured by western blot in MKN-74 and HGC-27 cells. **d** and **e** Proliferation of MKN-74 and HGC-27 cells was determined by MTT assay. **f** The protein level of Ki67 was measured by western blot in MKN-74 and HGC-27 cells. **g** Apoptosis of MKN-74 and HGC-27 cells was detected by flow cytometry assay. **h** and **i** Migration and invasion of MKN-74 and HGC-27 cells were assessed by transwell assay. *p < 0.05

To further investigate the interaction between circCNIH4 and DKK2, MKN-74 and HGC-27 cells were transfected with circCNIH4 or circCNIH4 + si-DKK2. The mRNA level of DKK2 was elevated by circCNIH4 and reversed by si-DKK2 in MKN-74 and HGC-27 cells, and the protein level of DKK2 exhibited a same trend (Fig. 4a, b). Subsequently, the expression of β-catenin was detected. The western blot result showed that the reduced protein level of β-catenin caused by circCNIH4 overexpression was rescued by silencing of DKK2 (Fig. 4c). Also, proliferation was inhibited in MKN-74 and HGC-27 cells transfected with circCNIH4 but restored when co-transfected with si-DKK2 (Fig. 4d, e). Ki67 was downregulated by overexpression of circCNIH4 and recovered by silencing of DKK2 in MKN-74 and HGC-27 cells (Fig. 4f). In addition, circCNIH4-induced apoptosis was reverted in MKN-74 and HGC-27 cells transfected with circCNIH4 + si-DKK2 (Fig. 4g). The inhibitory effects of circCNIH4 on migration and invasion of MKN-74 and HGC-27 cells were also abolished by silencing of DKK2 (Fig. 4h, i). These results signified that circCNIH4 inhibited Wnt/β-catenin pathway and gastric cancer cells progression via upregulating DDK2 expression.

### Knockdown of FRZB recused circCNIH4-mediated effects on gastric cancer cells

**Fig. 5:**
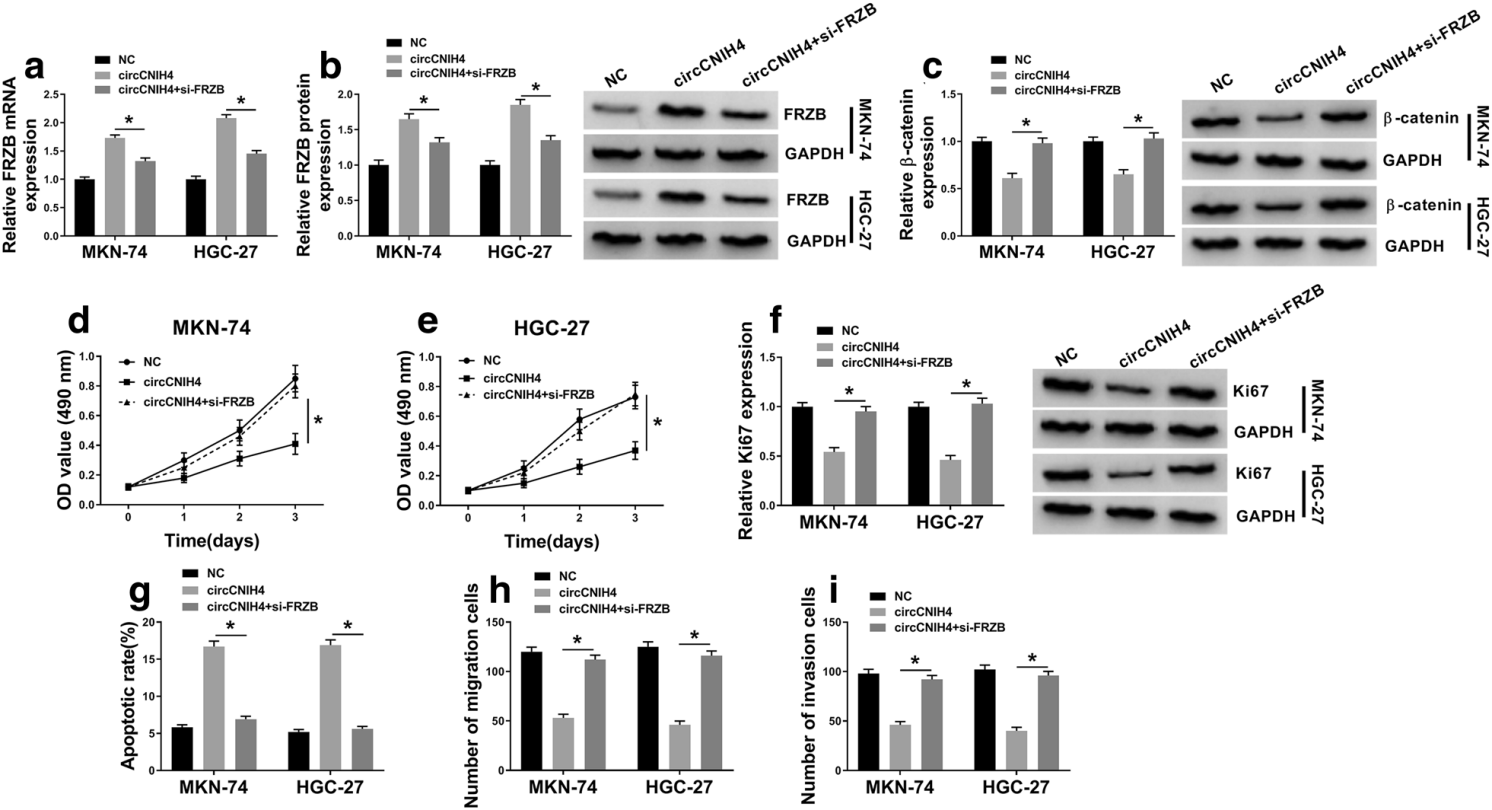
Silencing of FRZB reversed the effects of circCNIH4-mediated in gastric cancer cells. MKN-74 and HGC-27 cells were transfected with circCNIH4 or circCNIH4 + si-FRZB. **a** and **b** The mRNA and protein levels of FRZB were detected in MKN-74 and HGC-27 cells. **c** The protein level of β-catenin was measured by western blot in MKN-74 and HGC-27 cells. **d** and **e** Proliferation of MKN-74 and HGC-27 cells was determined by MTT assay. **f** The protein level of Ki67 was measured by western blot in MKN-74 and HGC-27 cells. **g** Apoptosis of MKN-74 and HGC-27 cells was detected by flow cytometry assay. **h** and **i** Migration and invasion of MKN-74 and HGC-27 cells were assessed by transwell assay. *p < 0.05

To further investigate the interaction between circCNIH4 and FRZB, MKN-74 and HGC-27 cells were transfected with circCNIH4 or circCNIH4 + si-FRZB. The mRNA and protein levels of FRZB were measured, and the results indicated that FRZB expression was upregulated by circCNIH4 and weakened by FRZB knockdown (Fig. 5a, b), while the protein level of β-catenin was downregulated by circCNIH4 and restored by si-FRZB in MKN-74 and HGC-27 cells (Fig. 5c). MTT assay showed that overexpression of circCNIH4 repressed proliferation of MKN-74 and HGC-27 cells, and this inhibitory effect was reversed by knockdown of FRZB (Fig. 5d, e). Besides, the protein level of Ki67 was decreased in MKN-74 and HGC-27 cells transfected with circCNIH4 and regained when transfected with circCNIH4 + si-FRZB (Fig. 5f). The apoptosis of MKN-74 and HGC-27 cells was drastically enhanced when circCNIH4 was overexpressed and recovered by FRZB knockdown (Fig. 5g). Additionally, the suppressed migration and invasion of MKN-74 and HGC-27 cells by circCNIH4 were also rescued by FRZB knockdown (Fig. 5h, i). These results revealed that circCNIH4 inhibited Wnt/β-catenin pathway and gastric cancer cells progression through regulating FRZB expression.

### Overexpression of circCNIH4 suppressed tumor growth through regulating DKK2 and FRZB expression

**Fig. 6:**
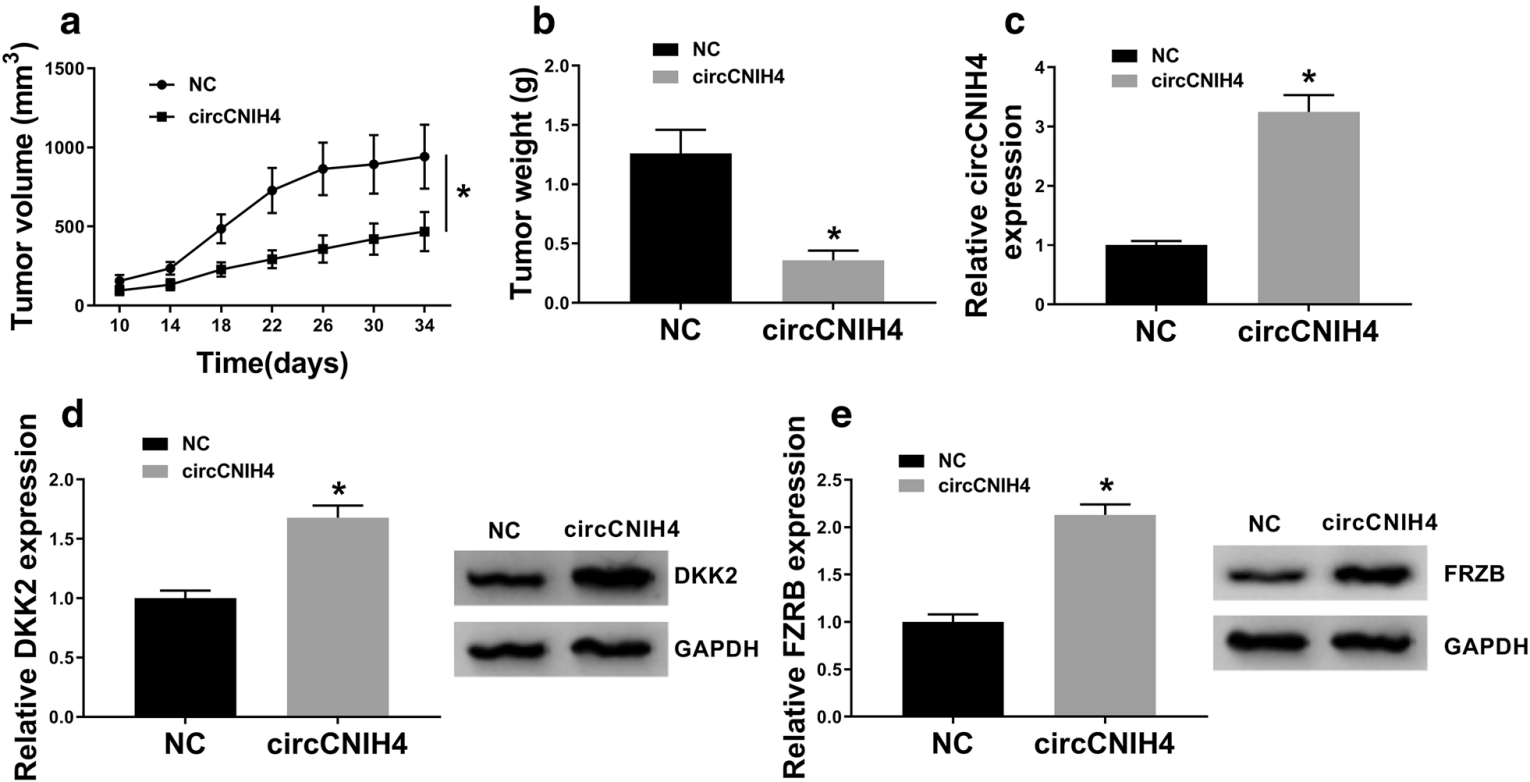
Overexpression of circCNIH4 inhibited tumor growth in gastric cancer in vivo. MKN-74 cells transfected with circCNIH4 or NC (empty vectors) were subcutaneously injected into nude mice. **a** Tumor volume was measured every 4 days after implantation for 14 days. **b** Tumor weight was measured at 34 days after implantation. **c** The expression of circCNIH4 was detected by qRT-PCR. **d** and **e** The protein levels of DKK2 and FRZB were detected by western blot. *p < 0.05

To verify the role of circCNIH4 in gastric cancer in vivo, the xenograft mouse model was utilized. The tumor volume and weight were measured, and the results manifested that tumor growth was significantly retarded in circCNIH4 group compared with NC group (Fig. 6a, b). Then qRT-PCR result showed that circCNIH4 was overexpressed in circCNIH4 group (Fig. 6c). Moreover, the protein levels of DKK2 and FRZB were also elevated in in circCNIH4 group (Fig. 6d, e). These data indicated that overexpression of circCNIH4 inhibited gastric cancer progression by regulating the expression of DKK2 and FRZB in vivo.

## Discussion

Gastric cancer is a common cancer with high mortality, which is a severe danger to human health. With the improvement of therapeutic strategy, the 5-year survival rate of gastric cancer patients in early-stage can reach more than 90% [3]. However, gastric cancer is usually diagnosed in the advanced stage. Understanding the molecular mechanism of regulating gastric cancer progression is of great significance for the development of early diagnosis and treatment of gastric cancer.

Emerging researches revealed that circRNAs participated in various physiological processes, such as cell proliferation, migration, invasion and differentiation [20–22]. Because of the circular structure, circRNAs have resistance to the digestion of RNA enzymes. Thereby they can act as applicable diagnostic biomarkers for human cancers [23]. CircCNIH4 has been shown to be downregulated in gastric cancer and may be a biomarker for the diagnosis of gastric cancer [10]. Besides, it was also proved that circCNIH4 was downregulated in multiple myeloma and located in the cytoplasm, and overexpression of circCNIH4 restrained proliferation and facilitated apoptosis in multiple myeloma [24]. In accordance with these studies, our data exhibited that circCNIH4 expression was lessened in gastric cancer tissues and cells. We also demonstrated that circCNIH4 was mainly located in the cytoplasm, and the functional experiments indicated that circCNIH4 impeded the development of gastric cancer in vitro and in vivo.

Wnt/β-catenin signal is well known to play a crucial regulatory role in tumor progression [25]. β-catenin is a key signaling transducing protein in the Wnt/β-catenin pathway. Normally, the β-catenin level is very low in the cytoplasm. When Wnt interacted with Frizzled, the Wnt signal was activated and β-catenin was cumulated in the cytoplasm, and then entered into the nucleus [26]. Intranuclear β-catenin combined with target genes and promoted their expression, thereby regulating the development of many cancers [27]. Numerous molecules have been reported to inhibit the activity of the Wnt/β-catenin pathway, such as SFRPs, DKK proteins, inhibition of the Dvl and Axin complex (IDAX), Wnt inhibitory factor 1 (WIF1) and so on [28–31]. In this study, overexpression of circCNIH4 reduced the expression of β-catenin, suggesting that circCNIH4 inhibited Wnt/β-catenin pathway in gastric cancer. Subsequently, the levels of certain Wnt antagonist genes were detected to explore the molecular mechanism of circCNIH4 on regulating the Wnt/β-catenin pathway. DKK2 and FRZB were found to be upregulated in both MKN-74 and HGC-27 cells after overexpression of circCNIH4.

DKK is a conserved secreted glycoproteins family, which contains four members, DKK1-4. DKK proteins have been reported to play an inhibitory part in Wnt/β-catenin signaling cascade [31]. DKK2 may serve as an inhibitor or activator of the Wnt/β-catenin pathway; its action may depend on the cellular context. For instance, DKK2 facilitated Wnt/β-catenin pathway and contributed to prostate cancer cells proliferation and invasion [32]. Overexpression of DKK2 repressed the progression of gastric cancer cells through Wnt/β-catenin pathway [33]. Similar to DKK2, FRZB also plays an inhibitory role in Wnt/β-catenin pathway in gastric cancer. It has been revealed that FRZB was upregulated in gastric cancer, and overexpression of FRZB impeded gastric cancer cell proliferation. Also, knockdown of FRZB enhanced the activity of β-catenin and promoted cell growth in gastric cancer [19]. In our study, the restoration experiments demonstrated circCNIH4 suppressed gastric cancer cell progression via elevating the expression of DKK2 and FRZB to repress Wnt/β-catenin pathway. This is the first study to elucidate the regulatory relationship between circCNIH4 and DKK2 or FRZB in gastric cancer; however, the precise molecular mechanism still needs to be further studied.

## Conclusion

In summary, our study showed that circCNIH4 was downregulated in gastric cancer and mainly located in the cytoplasm. Overexpressed circCNIH4 retarded proliferation, migration and invasion and promoted apoptosis via inactivating Wnt/β-catenin pathway in gastric cancer in vitro. Then, we found Wnt antagonist genes DKK2 and FRZB were upregulated by circCNIH4 overexpression in MKN-74 and HGC-27 cells and proved that circCNIH4 played its function by regulating DKK2 and FRZB expression and inactivating Wnt/β-catenin pathway in gastric cancer cells. Finally, the in vivo experiment indicated that circCNIH4 inhibited tumor growth via DKK2 and FRZB in gastric cancer. Our study provided a theoretical basis for the regulation of gastric cancer development, which might contribute to the diagnosis and treatment of gastric cancer.

## Supplementary Information

**Additional file 1: Figure S1.** The characteristics of the circCNIH4. **a** Divergent and convergent primers were used to validate the existence of circCNIH4 in MKN-74 and HGC-27 cells via RT-PCR. Divergent primers amplify circCNIH4 in cDNA but not in genomic DNA (gDNA), while circCNIH4 could be amplified by convergent primers in both cDNA and gDNA. The linear GAPDH was used as the negative control that could be amplified only by convergent primers in both cDNA and gDNA. Triangles connected at edge or angle represent divergent and convergent primers, respectively. **b** The splicing junction of circCNIH4 was validated by Sanger sequencing.

## Data Availability

Not applicable.
