# Supplementary figures and images for: CircCNIH4 inhibits gastric cancer progression via regulating DKK2 and FRZB expression and Wnt/β-catenin pathway
Source: J Biol Res (Thessalon). 2021 Aug 7;28:19. doi: 10.1186/s40709-021-00140-x (PMC8349030; doi:10.1186/s40709-021-00140-x)

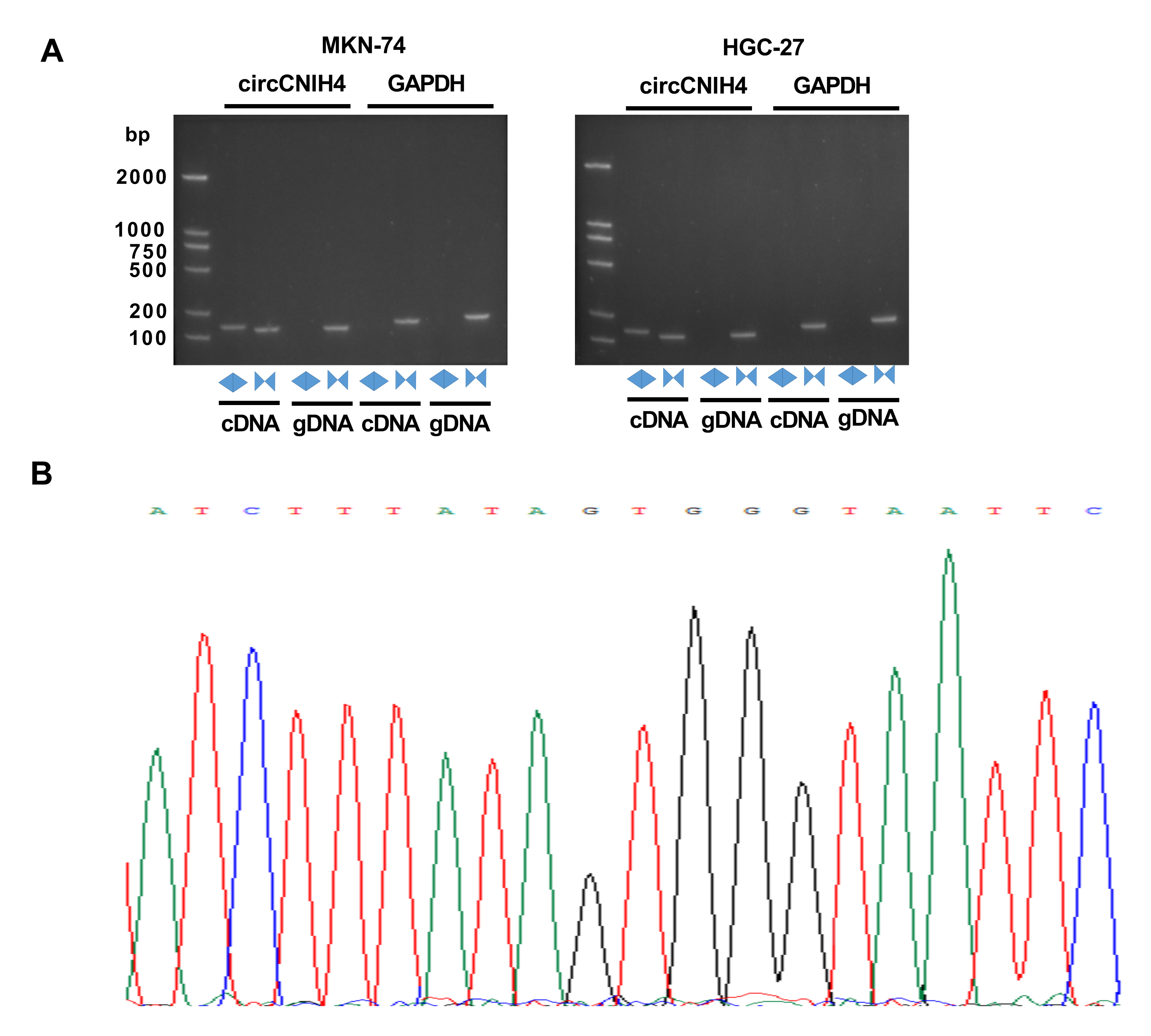

Supplement: Supplementary file 1 — Additional file 1: Figure S1. The characteristics of the circCNIH4. a Divergent and convergent primers were used to validate the existence of circCNIH4 in MKN-74 and HGC-27 cells via RT-PCR. Divergent primers amplify circCNIH4 in cDNA but not in genomic DNA (gDNA), while circCNIH4 could be amplified by convergent primers in both cDNA and gDNA. The linear GAPDH was used as the negative control that could be amplified only by convergent primers in both cDNA and gDNA. Triangles connected at edge or angle represent divergent and convergent primers, respectively. b The splicing junction of circCNIH4 was validated by Sanger sequencing. [file 40709_2021_140_MOESM1_ESM.tif]
